# Brain Atrophy in Relapsing Optic Neuritis Is Associated With Crion Phenotype

**DOI:** 10.3389/fneur.2019.01157

**Published:** 2019-11-01

**Authors:** Laura Navarro Cantó, Sara Carratalá Boscá, Carmen Alcalá Vicente, Sara Gil-Perontín, Francisco Pérez-Miralles, Jessica Castillo Villalba, Laura Cubas Nuñez, Bonaventura Casanova Estruch

**Affiliations:** ^1^Departament of Neurology, Hospital General Universitario de Elche, Alicante, Spain; ^2^Neuroimunology and Multiple Sclerosis Research Group, Hospital Universitari i Politècnic La Fe de València, Valencia, Spain; ^3^Neuroimmmunology Unit, Hospital Universitari i Politècnic La Fe, Valencia, Spain

**Keywords:** anti-MOG antibodies, CRION, optic neuritis, brain atrophy, biomarker

## Abstract

**Background and objective:** Chronic relapsing inflammatory optic neuritis (CRION) is one of the more common phenotypes related to myelin oligodendrocyte glycoprotein antibodies (MOG-Abs). The absence of specific biomarkers makes distinguishing between CRION and relapsing inflammatory ON (RION) difficult. A recent work has suggested a widespread affectation of the central nervous system in CRION patients. In order to search for a potential CRION marker we have measured brain atrophy in a cohort of patients, stratified by phenotypes: CRION, RION, multiple sclerosis with a history of optic neuritis (MS-ON), and MOG-Abs status.

**Methods:** A cross-sectional study was conducted in 31 patients (seven CRION, 11 RION, and 13 MS-ON). All patients were tested for MOG and aquaporin-4 antibodies (AQ4-Abs). Clinical data were collected. Brain atrophy was calculated by measuring the brain parenchyma fraction (BPF) with Neuroquant® software.

**Results:** Four of seven CRION patients and one of 11 RION patients were positive for MOG-Abs (*p* = 0.046) and no MS-ON patients tested positive to MOG-Abs. All patients were negative to AQ4-Abs. The BPF was lower in patients with CRION than patients with RION (70.6 vs. 75.3%, *p* = 0.019) and similar to that in MS-ON patients.

**Conclusions:** Brain atrophy in idiopathic inflammatory relapsing ON is present in patients with the CRION phenotype. Data from this study reflect that the optic nerve is a main target involved in these patients but not the only one. Our results should be further investigated in comprehensive and prospective studies.

## Introduction

Optic neuritis (ON) is a common manifestation in demyelinating diseases and the first symptom of MS in 15–20% of patients (1). The anti-aquaporin-4 antibody (AQP4-Ab) has been detected in 25% of patients who present with recurrent or simultaneous bilateral optic neuritis (2), and 3–5% of optic neuritis cases have a recurrent course in the absence of a neurologically or systemically-based disease (2), termed relapsing optic neuritis (RON). Two forms of RON have been described. Chronic relapsing isolated optic neuritis (CRION), originally described by Kidd, is used to refer to cases with an early response to corticosteroid treatment or a recurrence upon corticosteroid withdrawal or dose reduction (3), whereas RION is a non-progressive relapsing ON without steroid dependence (4–6). Although clinical indicators are helpful and are the main characteristics that distinguish RION from CRION (4–6), these patients are difficult to classify, especially due to the absence of specific biomarkers.

In the last decade, many studies have shown the presence of anti-myelin oligodendrocyte glycoprotein antibodies (MOG-Abs) in the serum of adults with acquired demyelinating syndromes of the central nervous system (CNS) (7–10). ON is the most frequent phenotype in MOG-related diseases (MOGrd) (7–13), and recent studies have suggested that these antibodies are specifically associated with the CRION profile (11–18). However, the clinical spectrum of MOG-Abs is broader and includes longitudinally extensive transverse myelitis (LETM), acute disseminated encephalomyelitis (ADEM), and brainstem syndrome, as described by the two longest studies (12, 13) and other studies (7–10). In addition, a recent paper reported two cases of encephalitis associated with MOG-Abs that were misdiagnosed as small-vessel CNS vasculitis due to biopsy results (19). Finally, MOG-Abs are rarely detected in the serum of adult patients with multiple sclerosis (MS) but have been found in a small subgroup of adult MS patients who usually exhibit atypical features, such as severe ON, severe and extensive myelitis, or brainstem involvement (20–22).

The criteria for a RON diagnosis include the absence of abnormalities in conventional MRI sequences. Based on the hypothesis that CRION may involve more than the optic nerve, we compared the degree of cerebral atrophy among patients with CRION (most of whom were seropositive for MOG-Abs), those with RION (only one with MOG-Abs), and patients with MS and a history of optic neuritis (MS-ON) in an attempt to identify CRION biomarkers. The clinical and paraclinical characteristics of these three groups were also compared.

## Methods

A total of 18 patients with relapsing optic neuritis (RON) were selected from the Neuroimmunology Unit of the University Hospital La Fe. The inclusion criteria were at least two episodes of ON or a simultaneous or rapidly sequential bilateral ON episode, or an episode of ON with early recurrence in the same eye upon removal of corticosteroids. Cases of ON with a non-inflammatory cause, cases associated with a systemic disease, and patients who fulfilled the diagnostic criteria for NMOSD were excluded (24). The patients were classified as having RION when there was an improvement and stability in visual function between relapses and as having CRION when there was steroid dependence and a greater degree of visual affectation disturbance. According to the McDonald 2010 criteria, 13 patients with MS and a history of ON were also included.

This study was cross-sectional, observational, and descriptive. A single face-to-face visit was performed in which the study was explained to the patient. All patients were informed and spontaneously consented to participate by signing a written agreement research form.

A neurological examination was performed, including an assessment of visual acuity, and blood samples were extracted. All patients were tested for antibodies targeting MOG and AQP-4. In this same visit, we retrospectively reviewed the clinical history of all patients with respect to disease type, recurrence status, visual acuity after the first episode, and treatment and laboratory and neuroimaging data were collected.

We compared the patients in three groups: CRION, RION, and MS-ON.

### Determinations

Sera from patients who agreed to donate sera and signed a specific informed consent form were stored at the Biobank of the Medical Research Institute of the Hospital La Fe (IISLaFe). Samples were aliquoted and stored at −80°C until determinations were made following legal and ethical requirements. This study was approved by the Ethic Committee of Investigation (CEIm La Fe).

### AQP4-Ab Detection

Indirect immunofluorescence (IFI) in cells transfected with AQP4 (EUROIMMUN Medizinische Labordiagnostika AG) was performed according to the manufacturer's protocol. Serum was diluted 1:10 in PBS-Tween.

### MOG-Ab Detection

**Figure 1:**
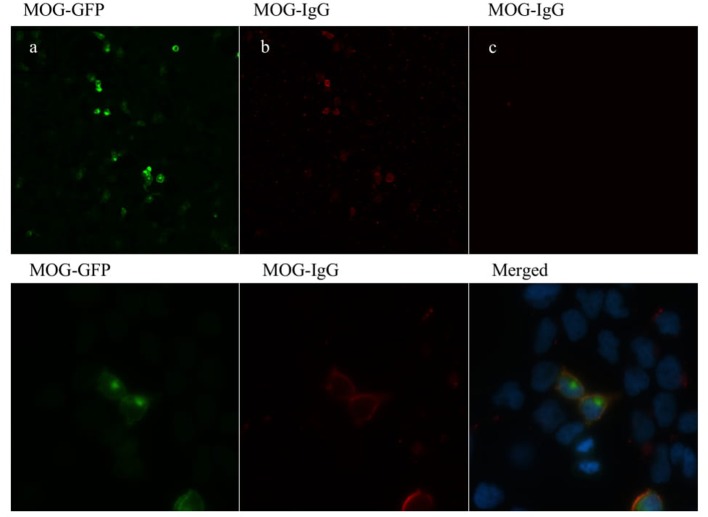
MOG-IgG antibodies detected by cell-based assay (CBA). HEK-293A cells expressing human full-length MOG-GFP (green) **(a)** following addition of MOG-IgG-positive RON serum sample **(b)** and negative MS serum sample **(c)** (titer of 1:160). MOG-IgG antibodies were visualized using a Cy3-conjugated goat anti-human IgG antibody (red). Images are shown in 20× (upper panel) and 100× (lower panel) magnification.

HEK (Human Embryonic Kidney) cells at a density of 50,000 cells/cm^2^ were transfected with an episome (pCEP4, Invitrogen, Walthman, MA, United States) containing a gene construct consisting of a fused full-length human myelin oligodendrocyte glycoprotein (MOG) gene (NM_206809.3) and GFP gene, with the latter in the C-terminal position. Predicted sublocalization (Target P 1.1 Server, www.ExPaSy.org) (25) showed that the transmembrane localization of the MOG protein was not altered by GFP gene fusion. HEK cells were transfected (Lipofectamine^(R)^ LTX & PLUS™ Reagent, catalog number 15338-100, Invitrogen) according to the manufacturer's protocol. Cells were then fixed with 4% paraformaldehyde, washed several times with PBS, and kept in PBS at 4°C for <1 week before processing. Cells were blocked with 1:200 goat IgG for 3 h at room temperature (15256, Sigma, St Louis–MO) and incubated with a 1:200 dilution of patient serum for an additional hour. After several washes, the cells were incubated for 1 h with a fluorescent pre-absorbed secondary antibody against human IgG (Cy3) (Abcam, ab97170, Cambridge, UK). Samples were mounted and observed under a fluorescence microscope (BX51 Olympus, Tokyo Japan), and 40× micrographs were taken from three replicates for each serum sample. At least 100 MOG-GFP-positive cells per replicate were analyzed for double staining, and counts were expressed as the percentage of double stained cells over MOG-GFP-positive cells (%DSC). This quantification served us as a titration method. Positive and negative controls from our cohort were kindly determined by a different laboratory with a distinct and validated technique (Dr. Saiz, Neuroimmunology Unit, Hospital Clinic Barcelona) and used as the gold standard. Commercial antibodies targeting MOG were also used as a positive control. At the established cut-off value (%DSC = 32; 2 units over mean plus SD), controls were classified as positive or negative with 100% concordance with the gold standard. MOG-IgG antibodies detected by cell-based assay are shown in Figure 1.

#### MRI Acquisition and Processing

MRI examinations were performed on a 3T MRI scanner. The MR imaging protocol included a 3D T1 magnetization-prepared rapid gradient-echo (MPRAGE) (TR = 2,600 ms, TE = 4.9 ms; voxel size = 1 × 1 × 1 mm^3^) without contrast. All brain MRIs were performed between June 2013 and June 2016. There were no significant differences in age when the MRIs were performed, and although the proportion of women was different among the three groups there were no differences between men and women for MRI measurements.

Brain atrophy was measured using NeuroQuant®. It performs an automated analysis that involved several steps, including stripping the skull and mapping the brain with a Talairach atlas. The global and regional volume data were calculated automatically in cm^3^ using Neuroquant® 3D software (25, 26). The software automatically calculates the total brain volume and brain parenchyma fraction (BPF) and provides values for each structure in the right and left hemispheres. Total cerebral volume was normalized for head size automatically by the software. For this study, we assessed the total brain volume, volume corresponding to the BPF, cerebral cortex volume, and thalamus volume. Neuroquant® automatically compares the resulting values with normative values.

Brain lesions were evaluated in T2-weighted sequences. Similarly, hyperintensity and edema of the optic nerve were also detected on T2-weight images and on Coronal Short Tau Inversion Recovery (STIR) images. Gadolinium enhancement of the optic nerve was evaluated with T1 post-contrast sequences. Spinal cord lesions were evaluated with sagittal T2W and STIR sequences.

### Statistics

SPSS v21.0 was used for the comparisons. The Mann-Whitney *U* test was used to compare continuous variables, and the Fisher exact test was used to compare frequencies and categorical variables. The level of significance was set at *p* < 0.05. Patients of the three groups were similar in distributions of age and gender (all *p* > 0.05). The calculation of the differences between groups of the volumetric measurements was made after adjusting for age, sex, and evolution time since first optic neuritis with multifactor ANOVA test (all *p* > 0.05).

## Results

Of the 18 patients with relapsing ON, seven were classified as CRION and 11 as RION. Four of the seven patients with CRION (57.1%) were positive for MOG-Abs and only one of the patients with RION (9.1%) was positive for MOG-Abs. All thirteen MS-ON patients were negative for MOG-Abs.

### Clinical and Demographical Characteristics of the Three Groups

The demographic and clinical characteristics of each group are summarized in detail in Table 1.

**Table 1:** Comparison among CRION, RION, and MS-ON patients.

	**CRION**	**RION**	**MS-ON**	***P*****-value (Mann-Whitney** ***U*** **test)**	
				**CRION vs. RION**	**CRION vs. MS-ON**
***n***	7	11	13		
**Sex F:M**	5:2	6:5	11:2	0.4^*^	0.4^*^
**Age of onset (y)**
Mean ±SD (min–max)	29.8 ± 13.7 (20.3–55.6)	30.2 ± 13.9 (7.6–56)	30.9 ± 5.4 (24–39)	0.9	0.4
**Disease duration (y)**
Mean ± SD	8.5 ± 6.1	10.4 ± 7.2	12 ± 4	0.5	0.2
Median (range)	6.70 (1.6–17.3)	9.3 (4.3–30.4)	10.3 (7.5–20.2)		
**Time to recurrence (m)**
Median (range)	9 (2–81.3)	14 (1.25–98.8)	11 (1.7–231)	0.9	0.7
**Number of ON attacks/patient**
Mean ± SD	4.4 ± 3.4	3.1 ± 2.2	1.8 ± 0.7	0.5	**0.046**
Median (range)	4 (1–11)	2 (1–9)	2 (1–3)		
**Simultaneous bilateral ON attacks;** ***n*** **patients (%)**	6 (85.7)	5 (45.5)	2 (15.3)	0.1^*^	0.004^*^
**>1 episode of ON** ***n*** **(%)**	6 (85.7)	11 (100)	9 (69)	0.4^*^	0.4^*^
**Steroid dependency;** ***n*** **(%)**	7 (100)	0	0	0.000^*^	0.000^*^
**VA after the first episode**
Mean ± SD	0.4 ± 0.2	0.6 ± 0.2	0.7 ± 0.3	0.069	**0.000**
Median (range)	0.5 (0.1–0.6)	0.6 (0.3–0.8)	0.7 (0.3–1)		
**VA at last follow-up**
Mean ± SD	0.5 ± 0.2	0.8 ± 0.1	0.9 ± 0.1	**0.003**	**0.000**
Median (range)	0.5 (0.1–0.7)	0.8 (0.6–1)	1 (0.6–1)		
**Last EDSS**
Mean ± SD	2 ± 0.8	1 ± 0.7	2.6 ± 1.5	**0.035**	0.4
Median (range)	2 (1–3)	1 (0–2)	2.5 (0–5.5)		
**OCB IgG;** ***n*** **(%)**	1 (14.3)	1 (9.1)	12 (92.3)	0.6^*^	0.001^*^
**MOG-Ab+;** ***n*** **(%)**	4 (57.1)	1 (9.1)	0	0.047^*^	0.007^*^
**AQP4-Ab+;** ***n*** **(%)**	0	0	0	NA	NA
**Abnormal orbital MRI;** ***n*** **(%)**	4 (57.1)	0	0	0.011^*^	0.007^*^
**Abnormal brain MRI;** ***n*** **(%)**	0	2 (18.2)	13 (100)	0.4^*^	0.000^*^
**Abnormal spinal MRI;** ***n*** **(%)**	0	2 (18.2)	6 (46.2)	0.4^*^	0.022^*^
**Number of treatments;** ***n***
Mean ± SD	2.2 ± 1.1	0.5 ± 0.9	2 ± 1.4	**0.003**	0.6
Median (range)	2 (1–4)	0 (0–3)	2 (1–4)		
**Treatment;** ***n*** **patients (%)**	4 (57.1)	2 (18.2)	9 (69.2)	0.1^*^	0.4^*^
**Last treatment (*****n*****, %)**
None	3 (42.9)	9 (81.8)	1 (7.7)		
AZA		1 (9.1)			
MFM	1 (14.2)				
RTX	3 (42.8)	1 (9.1)			
Any DMT			12 (92.3)		

* *Fisher exact test*.

*n, number; SD, standard deviation; F, female; M, male; y, years; m, months; ON, optic neuritis; BON, bilateral optic neuritis; VA, visual acuity; EDSS, Expanded Disability Status Scale; OCB, oligoclonal bands; AQP4-Ab, aquaporin-4 antibody; MOG-Ab, myelin oligodendrocyte glycoprotein antibody; AZA, azathioprine; MFM, mycophenolate mofetil; RTX, rituximab; IVIG, intravenous immunoglobulins; DMT, disease-modifying treatment; NA, not applicable. Values in bold when p < 0.05*.

Differences in the age of onset and gender distribution were not statistically significant between CRION patients and the other two groups (RION and MS-ON). In the RION group, almost half of the patients were men (45.5%), while in the other two groups there was a female predominance. Patients with MS had a longer disease duration and patients with CRION had the shortest disease duration (median: 10.3 vs. 6.7 years).

The patient with the largest number of neuritis relapses belonged to CRION group, with 11 relapses. The mean number of relapses per patient was significantly greater in the CRION group than in the MS-ON group (4.4 vs. 1.8, *p* = 0.046). Simultaneous bilateral involvement was characteristic of the CRION group (85.7%) and significantly higher than in MS-ON group (15.3%, *p* = 0.004). In the RION group, five patients suffered simultaneous bilateral ON. All patients with RION had a recurrent course. In the CRION group only one patient had monophasic course with right ON that tended to relapse following steroid withdrawal and therefore required long-term immunosuppression, whereas in the MS-ON group four patients had only one episode of ON. There were no significant differences in time of first recurrence. All patients with CRION showed steroid dependency, with recurrences in the dose reduction or withdrawal.

Visual acuity (VA) was significantly lower in CRION patients MS-ON patients, both after the first episode and in the last follow up (*p* = 0.000). Between the CRION and RION groups, the differences were significant for VA in the last follow up (*p* = 0.003), but they also showed a tendency toward significance after the first episode (*p* = 0.069).

MOG-Abs were detected significantly in more patients of the CRION group than in the group RION group (4 vs. 1, *p* = 0.047). All 13 MS-ON patients were negative for MOG-Abs. All patients (RON and MS-ON) were negative for the AQP4-Abs. Cerebrospinal fluid (CSF) was positive for oligoclonal band (OCB) in one patient of each group (RION and CRION).

All patients with MS had an abnormal brain MRI with typical brain lesions, compared with no patients in the CRION group (*p* = 0.000). Two patients in the RION group had non-specific T2 hyperintense lesions in white matter on brain MRI. Regarding orbital MRI, we found that about 60% of the CRION patients had T2-hyperintensity and gadolinium enhancement of the optic nerve, while no patient from the other two groups showed this alteration (*p* = 0.011 and *p* = 0.007, respectively). Spinal MRI was normal in all CRION patients and showed one subclinical chronic lesion in the cervical spine in two patients of the RION group who never developed any clinical symptoms of myelitis. Short transverse myelitis was detected in the spinal MRIs of six MS patients in relation to previous relapses.

There was a greater tendency to immunosuppress patients with CRION than with RION (57.1 vs. 18.2%), although this difference was not significant. Three CRION patients and one RION patient received long-term immunosuppression with rituximab. One of the CRION patients was treated with mycophenolate mofetil, and one RION patient was treated with azathioprine.

### Volumetric Characteristics of the Three Groups

Volumetric parameters are summarized in Table 2.

**Table 2:** Volumetric parameters.

	**CRION**	**RION**	**MS-ON**	***P*****-value (Mann-Whitney** ***U*** **test)**	
				**CRION vs. RION**	**CRION vs. MS-ON**
**Age at MRI**
Median (range)	38 (20–57)	34 (14–64)	45 (33–71)	0.8	0.3
**BPF, %**
Mean ± SD	70.6 ± 3.4	75.3 ± 3.4	71.4 ± 2.7	**0.019**	0.8
**TCV, cm**^**3**^
Mean ± SD	1,098.1 ± 39.3	1,232.7 ± 184.8	1,080.1 ± 127.2	0.2	0.056
**TCGMV**
Mean ± SD	434.9 ± 48.2	471.8 ± 75	439.3 ± 64.4	0.3	1
**TV**
Mean ± SD	14.4 ± 1.4	15.4 ± 2.6	13.5 ± 2.4	0.3	0.5

*M, months; BPF, brain parenchymal fraction; TCV, total cerebral volume; TCGMV, total cortical gray matter volume; TV, thalamic volume. Values in bold when p < 0.05*.

**Figure 2:**
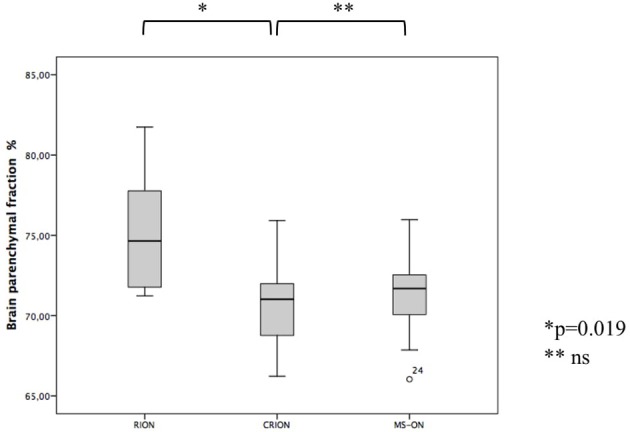
Differences in BPF among CRION, RION, and MS-ON patients (Mann-Whitney *U* test).

For the brain volume measurement, patients with CRION had a significantly lower BPF than patients with RION (70.6 vs. 75.3%, respectively, *p* = 0.019) (Figure 2). BPF was similar between CRION and MS-ON group (70.6 vs. 71.4%, respectively). Total cerebral volume was lower in the CRION and MS-ON group compared to the RION group, but this difference was not significant. Upon examination of the total cortical gray matter volume, the greatest amount of atrophy was detected in the CRION group, followed by that in MS patients and the RION group. Thalamic volume was lowest in patients with MS. These differences were not significant.

### Characteristics of MOG-Ab-Positive Patients

The clinical features of each patient positive for MOG-Abs are shown in Table 3.

**Table 3:** Clinical and radiological characteristics of MOG-ON patients.

**Patient**	**M/F**	**Age of onset (years)**	**Number of ON attacks (simultaneous BON attacks)**	**Steroid dependency**	**Phenotype**	**Neuroimaging**	**VFSS first ON**	**VFSS last follow-up**	**Treatment**
1	F	25	11 (1)	Yes	CRION	At onset: Optic nerve with gadolinium enhancement n At FU: normal	3	2	IVMP AZA (discontinued)
2	M	55	2 (2)	Yes	CRION	At onset: Optic nerve edema with gadolinium enhancement At FU: optic nerve atrophy	5	3	IVMP + PLEX RTX
3	F	29	3 (1)	No	RION	At onset: Normal At FU: Normal	3	1	IVMP
4	F	20	5 (1)	Yes	CRION	At onset: Normal At FU: Normal	4	2	IVMP MFM
5	F	20	1 (0)	Yes	CRION	At onset: Optic nerve with gadolinium enhancement At FU: Normal	5	2	IVMP RTX

*M, male; F, Female; BON, bilateral optic neuritis; VFSS, visual functional system score; FU, follow up; IVMP, intravenous methylprednisolone; AZA, azathioprine; MFM, mycophenolate mofetil; RTX, rituximab*.

The majority (80%) of the five patients with MOG-Abs positivity were women (80%), with a median age of onset of 25 years (range 20–55). All but one had a relapsing course. A total of 22 attacks were recorded for all patients, with a mean recurrence of 46 attacks (median 3, range 2–11). Four patients (80%) exhibited simultaneous bilateral involvement of both optic nerves, with one patient experiencing this twice. A total of 80% (4/5) of the MOG-Abs-positive patients exhibited steroid dependency. These patients were classified as having CRION, and only one was classified as having RION (CRION:RION ratio of 4:1). The first ON attack led to a VA lower than 20/60 in four patients, with a bad final visual outcome in one patient and complete recovery in one patient.

Three patients presented with a T2-hypersignal and gadolinium enhancement of the optic nerve on the orbital MRI. No brain lesions were detected in any patient. All MOG-Ab-positive patients were treated with 1 g of intravenous methylprednisolone (IVMP) per day for 5 days in the acute phase; the four CRION patients received a pattern of descending oral prednisone after a megadose. One patient also received plasma exchange due to partial recovery after intravenous corticosteroids. Three of the five patients remained immunosuppressed at the time of the study, two with rituximab and one with mycophenolate mofetil. Of the two remaining patients, the RION patient only received treatment with corticosteroids in the acute phase, and the other had received azathioprine but did not present new episodes upon withdrawal (Table 3).

## Discussion

In this study, patients with the CRION phenotype had a significantly lower BPF than patients with RION. More than half of patients with the CRION phenotype were positive for MOG-Abs (57.1%) and only one patient in the RION group (9.1%) was positive. Most patients with MOG- Abs have the CRION phenotype (80%). To our knowledge, this paper is the first to provide data on brain atrophy in patients with relapsing ON with MOG-Abs. Distinguishing between the diagnosis of RION or CRION is difficult, because there are no biomarkers that facilitate the diagnosis of these entities (4, 6). The greater amount of cerebral atrophy found in CRION patients than in RION patients suggests that cerebral atrophy could be a biomarker of CRION and could help to differentiate CRION from RION.

The presence of brain atrophy is indicative of the surrounding pathological condition responsible for the widespread damage of the CNS. Therefore, brain atrophy has been used as a surrogate marker of neurodegeneration (28). In demyelinating diseases, the presence of brain atrophy has been demonstrated in MS (29) and NMOSD, being more severe in the former (27).

The only work that studied the impact of CRION on the brain was published by Colpak et al. (23), who studied changes in the apparently normal white matter in a group of six CRION patients using DTI. These authors demonstrated widespread effects in the brain that implicated not only the visual tract, as was expected due to the Wallerian degeneration, but also the cerebellum, right superior cerebellar peduncle, body and splenium of the corpus callosum, left thalamus, posterior cingulum, and bilateral posterior bundles of the inferior fronto-occipital tracts, and the inferior longitudinal fasciculi, which exhibited increased fraction of anisotropy (FA) and decreased radial diffusivity (RD). Interestingly, the main finding of this study in both the optic pathways and the optic chiasm was an increase in FA and a decrease in RD, which is the opposite of what occurs in demyelinating lesions. However, other white matter areas exhibited changes suggestive of inflammation or demyelination. Therefore, these authors concluded that, in the brains of these patients, a combination of pathological processes seemed to be produced, supporting the hypothesis that CRION is a disorder independent from MS or NMOSD (23).

We compared the characteristics of relapsing optic neuritis with CRION and RION phenotypes and those of MS-ON patients. All MS patients were negative for MOG-Abs, as reported by other studies (11, 31). The CRION patients showed a higher number of ON attacks, frequency of recurrence, and bilateral involvement than the other two groups, but these differences were only significant when compared to the MS-ON group. In contrast to sequential bilateral ON, simultaneous bilateral ON is not a typical finding in MS. All these results are consistent with those of other studies (11, 18, 31–33).

As in the natural history of patients with MS or NMOSD, not all patients with CRION meet all diagnostic criteria from the beginning as the disease is a dynamic process. In our study, the first episode of one CRION MOG-Ab-positive patient was followed by almost 7 years before the development of CRION, and the first episode of a seronegative RION patient was followed by almost 8 years before the development of RION. This is similar to what was reported by the series by Waschbisch et al. (16). After an episode of ON, in addition to an exclusion study that includes screening for autoantibodies, a long follow-up is necessary due to the possibilities of a conversion to MS or NMOSD or progression to a CRION phenotype.

Additionally, we have reported the detailed clinical features of a series of 5 MOG-Abs-positive patients and relapsing ON without other symptomatology. Similar to previous reports (14–18, 30, 31, 34), in our study, the steroid dependency and the CRION phenotype were associated to MOG-Abs. The duration of the disease of our patients was long, similar to the follow-up of the Jarius et al. (13) cohort (80.4 months in our cohort and 75 months in the German cohort) and revealed that relapses were confined to the optic nerves in all patients. Of the four patients classified as having CRION in our series, 3 clearly fulfilled the diagnostic criteria for CRION as proposed by Petzold and Plant (4). The patient who did not meet all the criteria did not show gadolinium enhancement of the optic nerve on an MRI. Despite the criteria proposed by Petzold and Plant (4), most authors (6, 14–16, 32) agree that steroid dependence is the only fundamental characteristic that distinguishes CRION from RION, as Kidd et al. (3) originally described, and MRI findings are not essential. For other authors, such as Benoilid et al. (2), CRION patients are defined by a progressive decrease in visual acuity between relapses.

In a recent paper, Lee et al. (32) investigated the presence of MOG-Abs in patients who met current CRION diagnostic criteria and observed that patients with a RION phenotype without steroid dependency were not positive for MOG-Abs, as was observed in our group of RION patients.

The phenotypes of MOGrd have been shown to extend to more aggressive and diffuse forms than previously thought (7–10). MOGrd include cases from acute demyelinating encephalomyelitis to severe forms of recurrent encephalopathy with vasculitis (19). At the moment, the origin of brain atrophy in this limited type of MOGrd affecting the optic nerve is unknown; the retrograde Wallerian degeneration of the optic tract and the occipital lobes as a consequence of retinal damage could explain some degree of brain atrophy, but the widespread effects of other regions in the CNS, as demonstrated by Colpak et al. (23), suggests a more profound effect on MOGrd. The pathogenic mechanisms that lead to this atrophy are unknown.

Prolonged treatment with pulsed IVMP slows the rate of whole-brain atrophy progression (35, 36), however short-term effect of IVMP may contribute to reduction of brain volume especially in the first 2 months (37). All CRION patients in this series had steroid dependency but only one received treatment with corticosteroids in the 3 months prior to the MRI. The median time from the last relapse to the MRI in the group of CRION patients was 17 months.

This study is limited by various factors, mainly related to our small sample, as well as the transversal, observational, and retrospective design of the study. Brain MRI was not performed at the beginning or at the same time in all patients, but the absence of significant differences between the groups suggests that cerebral atrophy is related to pathogenic mechanisms and not to the time of evolution. In fact, the CRION group, which was the one with the highest degree of brain atrophy, also had the shortest evolution time until MRI was performed.

*In vivo* measures complimentary to our volumetric MRI data, such as optical coherence tomography (OCT), would have helped to better document our results. Furthermore, volumetric measurements of the occipital lobe would also be an interesting addition to this data.

In summary, in addition to confirming that MOG-Abs are frequently associated with the CRION, our findings also suggest, for the first time, that brain atrophy is related to the CRION phenotype. Data from this study reflect that the optic nerve is a main target involved in these patients but not the only. Our results must be confirmed by more comprehensive and prospective studies.

## Data Availability Statement

The datasets generated for this study are available on request to the corresponding author.

## Ethics Statement

The studies involving human participants were reviewed and approved by Ethic Committee of Investigation (CEIm La Fe). Written informed consent to participate in this study was provided by the participants' legal guardian/next of kin.

## Author Contributions

LC: design and conceptualized study, analyzed the data, and drafted the manuscript. SB: acquisition of volumetric data. CV, SG-P, and FP-M: assistance on the data acquisition and review the manuscript. JV and LN: acquisition of laboratory data. BC: study supervision, interpretation, drafting, reviewing the manuscript, and Appendix.

### Conflict of Interest

The authors declare that the research was conducted in the absence of any commercial or financial relationships that could be construed as a potential conflict of interest.

## Appendix Authors

**Table:**

**Name**	**Location**	**Role**	**Contribution**
Laura Navarro	Hospital General Universitario de Elche	Author	Designed and conceptualized study; analyzed the data; drafted the manuscript
Sara Carratalá	Hospital Universitari i Politècnic La Fe de València	Author	Acquisitioned volumetric data
Carmen Alcalá	Hospital Universitari i Politècnic La Fe de València	Author	Assisted data acquisition and reviewed the manuscript.
Sara Gil-Perontín	Hospital Universitari i Politècnic La Fe de València	Author	Assisted data acquisition and reviewed the manuscript.
Francisco Perez-Miralles	Hospital Universitari i Politècnic La Fe de València	Author	Assisted data acquisition and reviewed the manuscript.
Jéssica Castillo	Hospital Universitari i Politècnic La Fe de València	Author	Acquisitioned laboratory data
Laura Cubas	Hospital Universitari i Politècnic La Fe de València	Author	Acquisitioned laboratory data
Bonaventura Casanova	Hospital Universitari i Politècnic La Fe de València	Author	Study supervision, interpretation, drafting, and reviewing the manuscript

*LN conceptualized and designed the work. PN, LM, FM, MK, CS, NM, MP, MS, and PB-N acquisitioned, analyzed, or interpreted data for the work. PN, MP, MS, and PB-N drafted the work. All authors were involved in the critical revision of the manuscript for important intellectual content*.
